# Education Research: Embracing the Unknown—Introducing Clinical Uncertainty Into the Neurology Clerkship Curriculum

**DOI:** 10.1212/NE9.0000000000200220

**Published:** 2025-06-09

**Authors:** Aleksandra Safonova, Ashley Paul, Doris G. Leung, Andres Fernandez, Dimitrios Papanagnou, Rachel Marie E. Salas

**Affiliations:** 1 Neurology, Johns Hopkins University, Baltimore, MD;; 2 Neurology, Thomas Jefferson University, Philadelphia, PA; and; 3 Thomas Jefferson University, Philadelphia, PA.

## Abstract

**Background and Objectives:**

The field of neurology has inherent uncertainty, and there is currently a limited focus on how to navigate uncertainty in formal neurology training. This is an integral issue as uncertainty has been shown to correlate with increasing clinician burnout and overall negative health care outcomes. The aim of this study was to address this gap by introducing diagnostic uncertainty using case-based learning during the neurology clerkship. The objective of the study was to compare medical students' perceptions of clinical uncertainty before and after the course.

**Methods:**

Second-year, third-year, and fourth-year medical students participated in a 60-minute case-based learning exercise incorporating uncertainty held at the start of the neurology clerkship. Students were given an optional precourse and postcourse survey incorporating the clinical practice uncertainty domains (CPUDs). Data were collected and analyzed from August 2023 to June 2024.

**Results:**

Eighty-eight medical students participated in this course. There was a statistically significant increase in agreeability to several CPUDs in the postcourse survey compared with the precourse survey, including “I feel prepared to address uncertain situations during clinical clerkships” and “I am confident in my ability to communicate to patients during clinical situations that may be uncertain.” Most of the students who responded to the postcourse survey found this session useful.

**Discussion:**

Implementation of a standardized neurology curricular course focused on uncertainty is feasible and potentially valuable. There are several limitations to this study including the use of a postpositivist approach with the use of quantitative methods and the short follow-up period.

## Introduction

Uncertainty is endemic to the field of neurology. For one, the functions of the brain remain largely unknown, limiting the complete pathophysiologic understanding of disease. Each brain is also remarkably different, affected by variations in neurodevelopment and neuroplasticity throughout life. In addition, neurologic symptoms such as headache and numbness are often subjective, and there can be significant overlap in the presentation of disorders such as stroke, migraines, and seizures.^1^ Biomarkers are not yet widely available for many progressive neurodegenerative disorders, making diagnosis particularly challenging.^2^ These uncertainties further complicate neuroprognostication, which is a common neurologic consultation by families and medical teams for a variety of disorders ranging from strokes to comas.

Medical students, residents, and clinicians consistently rate neurology as one of the most difficult specialties and report having the least confidence in diagnosing and treating neurologic problems.^3,4^ Given the complexity of neurology, this in turn can be a driving factor for increasing clinical uncertainty. Unfortunately, there is a limited focus on how to navigate uncertainty in formal neurology training. However, this issue is integral as uncertainty has been shown to correlate with increasing clinician burnout, as well as resource overutilization, poor patient-physician relationships, and overall negative health care outcomes.^5,6^ Neurology especially has been shown to have higher rates of burnout and lower satisfaction with work-life integration compared with other specialties.^7-9^ Several studies show that this is partly due to limitations in diagnosis and treatment; for instance, the challenge of treating neurologic disorders has been described as “therapeutic nihilism.”^10^ This suggests a need for standardization of recognition and management of uncertainty in neurology. To fill this gap, we created and implemented an uncertainty activity for our neurology clerkship.

Renee Fox underscored the prevalence of medical uncertainty through recounting her first-hand experience with medical students in the 1950s. “What students found particularly ‘disquieting’ were those medical situations in which problems of uncertainty and problems of meaning were joined.”^11^ Since then, there have been numerous definitions and frameworks attempting to delineate different types of medical uncertainty. One of the most expansive taxonomies focused on sources of uncertainty (probability, ambiguity, and complexity), issues (scientific, practical, and personal), and the affected locus (patient, physician).^12^ Others have proposed frameworks for diagnosing and managing uncertainty. The Cynefin framework, initially created for decision making in economics and subsequently adapted to the medical field, categorizes clinical situations into 4 domains of increasing uncertainty (simple, complicated, complex, and chaotic). Each domain requires different approaches for management of uncertainty.^13,14^ Another framework^15^ outlines a model to help clinicians communicate uncertainty with patients by acknowledging “the certainty of uncertainty,” creating multistep plans with built-in contingencies, and using shared decision making with the patient. The latter 2 frameworks were used in our curriculum as will be described later. In parallel with the expansion of the field of medical uncertainty, there has been a shift in the paradigm of current models of training in medical education. While previous models focused on reinforcing certainty through multiple-choice questions and structured clinical examinations, there is now a transition to incorporate uncertainty training into the medical school curriculum to encourage recognition of uncertainty, share strategies to deal with uncertainty, and facilitate communication with patients and families during times of uncertainty. These skills are vital components of a physician's practice, as outlined by the most recently published AAMC's physician set of competencies: “Recognize that ambiguity is part of clinical health care and respond by utilizing appropriate resources in dealing with uncertainty.”^16^

While there have been several studies exploring the perception of medical uncertainty among medical students and the approaches to introduce these conversations into a curriculum, there is significant variability. One study found that team debriefs, role plays, case-based and team-based learning, and sharing narratives were useful regarding preparing students for uncertainty.^17^ Alternatively, another found that a curriculum with a case-based simulation focused on uncertainty led to students expressing confusion regarding their role and feelings of “inaction.”^18^ Additional studies have investigated the use of other humanity-based programs such as reflective learning diaries and philosophical courses.^19,20^ A review of uncertainty in medical training by Patel et al. found that problem-based and humanity-based were the most common modalities of uncertainty education, and that students found the training valuable for their longer term development of uncertainty tolerance.^21^

Although there are numerous studies exploring recognition and management of uncertainty in the medical school curriculum as well as various specialties, including emergency medicine,^22^ oncology,^23^ and primary care,^24^ there are limited studies exploring uncertainty in neurology, a field with inherent diagnostic uncertainty. The goal of this study was to incorporate clinical cases into a neurology clerkship and evaluate its impact on students' perception of uncertainty.

## Methods

### Curriculum Description

The activity was integrated into the neurology clerkship over the course of the 2023–2024 academic year. This included approximately 10 students per cycle. A 60-minute case-based learning exercise was held at the start of the neurology clerkship. Each session was led by a neurology resident, and sessions included second-year, third-year, and fourth-year medical students rotating on the neurology clerkship. One uncertainty case was discussed per session. Students participated in history taking, formulating a differential diagnosis, ordering diagnostic tests, reframing the differential based on results of tests, and working through the management and discussion of uncertainty with the patient. The Cynefin framework was included throughout the cases to identify different types of uncertainty, as well as other models for management of uncertainty and patient discussion as outlined above. The cases and framework were adapted from Papanagnou et al.^17^ There was no control group because this was meant to be an equitable experience to all medical students.

### Case Development

Three patient scenarios were designed by a neurology resident with input from the participating faculty coauthors and were based on the most common neurologic diagnoses seen by inpatient neurologic consult services, including altered mental status, seizure, and headache.^25^ The cases did not have a singular correct diagnosis at the end. The cases were reviewed and approved by researchers involved in medical education, and a pilot session was conducted with 3 fourth-year medical students to ensure appropriate learning content for medical students completing the Neurology Core Clerkship.

#### Study Design

We used a postpositivist approach with quantitative methods using surveys because this has been validated in previous research studies evaluating uncertainty in medical education. Students were invited to complete an optional survey distributed through email before each session started and at the end of their month-long neurology clerkship. The precourse surveys included clinical practice uncertainty domains (CPUDs), which consist of a series of 4 statements regarding reactions to uncertainty in clinical scenarios, including “I feel prepared to address uncertain situations during clinical clerkships,” “I am confident in my ability to communicate to patients during clinical situations that may be uncertain,” “When I encounter clinically uncertain situations, I am still able to form meaningful relationships with my patients,” and “When I encounter clinically uncertain situations, my well-being is negatively affected.”^16^ The postcourse surveys contained the same clinical practice uncertainty domains. Both surveys have been validated and used in previous educational research studies. The scores for both surveys were obtained using a five-point Likert scale ranging from “1 = completely disagree” to “5 = completely agree.” The postcourse survey evaluates the course's impact using Kirkpatrick level 2a.^26^

#### Analysis of Data

Statistical analyses were performed using GraphPad Prism 9.0.0. Composite survey results scored on Likert scales were considered as non-normally distributed continuous variables. Wilcoxon rank-sum tests were performed to assess hypotheses, with statistical significance considered at *p* < 0.05. Correlation analyses were performed using Spearman rank correlation tests. Chi-squared tests were performed to assess statistical significance for precourse and postcourse surveys. To assist in the analysis given a small number of students may choose one specific category, “agree” and disagree” comments were aggregated.

### Standard Protocols Approvals, Registrations, and Participant Consents

This study was reviewed by the Institutional Review Board (IRB) at the Johns Hopkins Hospital and was exempt from convened IRB review. The need for consent was waived (IRB number: CIR00106067).

### Data Availability

Data will be made available on request directed to the corresponding author.^24-26^

## Results

### Demographic Data

Eighty-eight medical students between August 2023 and June 2024 completed the precourse survey (100% response rate), and 61 medical students completed the postcourse survey (69.3% response rate). A total of 59.1% were female, and 38.7% were male. The mean age was 26 years, with a median of 25 and a range from 23 to 39 years of age.

The precourse and postcourse scores for the CPUDs were evaluated. For the CPUD “I feel prepared to address uncertain situations during clinical clerkships,” there was a statistically significant increase in agreeability in the postcourse survey, with the precourse survey showing that 33.7% of students responded with either 1 or 2 on the Likert scale and 38.4% responded with either 4 or 5, and the postcourse survey showing that 0% of students responded with either 1 or 2 and 75% responded with either 4 or 5 (*p* < 0.0001). For the CPUD “I am confident in my ability to communicate to patients during clinical situations that may be uncertain,” there was also a statistically significant increase in agreeability in the postcourse survey, with the precourse survey showing that 16.1% of students responded with 1 or 2 and 49.5% responded with either 4 or 5, and the postcourse survey showing 1.67% of students responded with a 1 or 2 and 75% responded with a 4 or 5 (*p* = 0.0006) (Figure). There was not a significant difference in responses for the other 2 CPUDs—“When I encounter clinically uncertain situations, I am still able to form meaningful relationships with my patients” and “When I encounter clinically uncertain situations, my well-being is negatively affected.” 71.4% of students who responded to the postcourse survey found this session useful.

**Figure:**
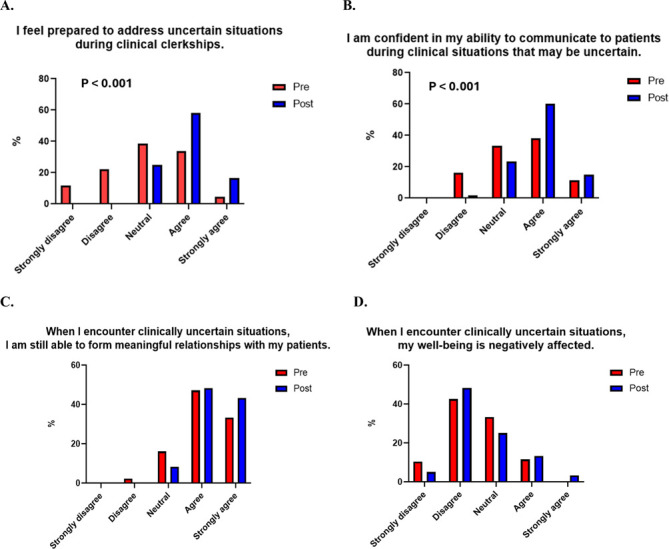
Pre- and Post-Course Clinical Practice Uncertainty Domain Scores The figure shows the pre- and post-distribution of levels of agreement on the Likert scale in each of the 4 elements of the clinical practice of uncertainty domains. There was a statistically significant increase in agreeability to feeling prepared to address uncertainty during clinical clerkships as well as feeling confident communicating with patients in uncertain clinical situations. There was not a significant difference regarding ability to form meaningful relationships with patients and negative effect on well-being.

## Discussion

This activity was created to prepare medical students for the clinical uncertainty they are likely to experience in practice in the context of case-based simulations centered around the concept of uncertainty. This was introduced at the beginning of the neurology clerkship for second-year, third-year, and fourth-year medical students, with the goal of providing a framework for recognizing and communicating uncertainty before starting clinical rotations. We found that there was an increase in students' perceived comfort and preparedness for addressing uncertainty after the course. This suggests a potential benefit to recognition of uncertainty and intervention in this field. In addition, most of the students who responded to the postcourse survey found this course useful. This is a valuable learning point as it shows the implementation of a standardized curricular course like the one outlined here is feasible and potentially valuable.

There were limitations to this study. While the purpose of the course was to help students recognize and communicate uncertainty, it is difficult to elicit a sustained change in students' perceptions and clinical practice through a 1 hour-long session. The precourse and postcourse surveys represented a subjective outcomes-based measure assessed by the students although it is unknown whether the changes perceived by the students after the course remain present over time. In addition, a follow-up time frame of one month is relatively short. It would be useful to evaluate the impact of this course throughout medical school, residency, and subsequent clinical practice. However, a challenge is the inherent difficulties with follow-up and student retention as can be seen in this study as well: the precourse survey had a 100% response rate because time was given in person during the session to complete the survey. The lower postcourse survey response rate is likely attributable to survey fatigue and asynchronous data collection. Future iterations may benefit from in-person opportunities to complete the postcourse survey. Given the complexity of this topic, it is inherently difficult to measure outcomes through a survey-based approach. This is a major limitation as it takes a postpositivist approach to studying uncertainty with quantitative methods, whereas a constructivist approach with qualitative methods would provide a deeper understanding of uncertainty. One of our future steps includes the addition of a focus group at the end of the course. Other potential options for further exploration include the use of simulations or evaluation during the clerkship in real-life scenarios. As emphasized earlier in this article, uncertainty is prevalent throughout all disciplines of medicine and affects physicians throughout their clinical practice. There is a growing need for standardized approaches to introduce uncertainty into medical education through various specialties and perspectives.

Our curricular intervention aimed to address this gap and represents a feasible and valued part of the neurology clerkship. Further investigation is warranted to evaluate the effect of introducing an educational activity focused on uncertainty in the neurology setting.

## Glossary

CPUDs: clinical practice uncertainty domains
